# Tricuspid annular plane systolic excursion/pulmonary arterial systolic pressure ratio as a predictor of in-hospital mortality for acute heart failure

**DOI:** 10.1186/s12872-022-02857-6

**Published:** 2022-09-17

**Authors:** Mohamed Naseem, Amr Alkassas, Ahmed Alaarag

**Affiliations:** Cardiovascular Medicine Department, Tanta Faculty of Medicine, Tanta University Hospital, Tanta, 31511 Egypt

**Keywords:** TAPSE/PASP ratio, In-hospital mortality, Acute heart failure

## Abstract

**Background:**

Right ventricular (RV) function is an important prognostic factor in heart failure. Patients with impaired right ventricular function have a poorer prognosis. The ratio between a tricuspid annular plane systolic excursion (TAPSE) and pulmonary artery systolic pressure (PASP) is a simple non-invasive parameter that has shown a good correlation with invasively estimated right ventricle (RV)-pulmonary artery (PA) coupling. The current study aimed to determine the value of the non-invasive evaluation of RV-PA coupling using the TAPSE/PASP ratio in predicting in-hospital mortality in patients with acute heart failure.

**Methods:**

We included 200 patients with (heart failure and reduced ejection fraction) HFrEF presented by acute heart failure. Echocardiographic evaluation for left ventricle systolic and diastolic function was performed at the time of admission. RV functions were evaluated by calculating the following (TAPSE, PSAP, TAPSE/PASP ratio). Data were analyzed to find the predictors of in-hospital mortality.

**Results:**

The study cohort included two hundred consecutive patients who were hospitalized for a diagnosis of acute decompensation of chronic heart failure. The in-hospital mortality rate was 12%. TAPSE/PASP was an independent predictor for in-hospital mortality (odd ratio = 3.470; 95% confidence interval, 1.240–9.705, *p*-value = 0.018) and (odd ratio = 18.813; 95% confidence interval, 1.974–179.275, *p*-value = 0.011) in univariate and multivariable logistic regression analyses respectively. In ROC curve analysis, TAPSE/PASP with a cut-off value < 0.4 mm/mmHg had a sensitivity of 79.17, a specificity of 47.73, and an area under ROC curve = 0.666 for predicting in-hospital mortality.

**Conclusions:**

The non-invasive TAPSE/PASP ratio could be an independent predictor of mortality in HErEF patients presenting with acute heart failure.

## Background

Acute heart failure (AHF) has a poor prognosis, with a dramatic increase in mortality and rehospitalization [1, 2]. The in-hospital mortality of patients with AHF was reported to range from 4 to 11% in most published AHF registries [3–5] and quite high post-discharge rehospitalization rates at 60- to 90-day from 25 to 30% [6].

In heart failure patients, right ventricular (RV) function is an important prognostic factor. Patients with impaired (RV) function have a poorer prognosis than those with normal RV systolic function with a two- to threefold increase in the risk of cardiac death, regardless of the degree of left ventricular (LV) dysfunction [7, 8].

Pulmonary hypertension (PH) is the most important cause of right ventricular failure (RVF) in heart failure patients [9], with both PH [9] and RVF being related to worsening clinical outcomes in heart failure (HF) patients [7, 10].

The evaluation of tricuspid annular plane systolic excursion (TAPSE) is a simple, and non-invasive method for assessing the RV systolic function in patients with heart failure [11, 12].

Maintaining right ventricular -pulmonary artery coupling refers to the ability of right ventricular contractility to compensate for increased afterload [13].

The gold standard for assessment of right ventricular-pulmonary artery (RV-PA) coupling requires invasive recordings of pressure–volume loops for measurement of the end-systolic/arterial elastance (Ees/Ea) ratio [14, 15].

Echocardiographic measurement of the ratio between (TAPSE) and pulmonary artery systolic pressure (PASP) is a simple noninvasive parameter that has shown a good correlation with invasively estimated RV-PA coupling [15].

A lower TAPSE/PASP ratio (indicating poor RV-PA coupling) has been associated with adverse prognoses in patients with cardiovascular disease [15–22].

The present study aims to determine the value of the TAPSE/ PASP ratio in predicting in-hospital mortality in patients with acute heart failure.

## Methods

### Study population

The present study prospectively included 200 consecutive patients aged ≥ 18 with acute decompensation of a previously known HF and reduced ejection fraction (HFrEF), admitted to the Cardiology department, Tanta University between January 2021 till December 2021.

HFrEF was defined as HF with a (left ventricular ejection fraction) LVEF < 40% [22]. Acute decompensated heart failure was defined as worsening HF symptoms resulting in the need for unplanned emergency department (ED) visits, or hospitalization. The diagnosis of prior HF was based on history, echocardiography, radionuclide studies findings, and patients’ medical records.

Patients' New York Heart Association Functional (NYHA) functional class were evaluated depending on the history taken from patients about their condition in the last 2 weeks before admission.

Patients with acute coronary syndrome, myocarditis, congenital heart disease, and cardiogenic shock were excluded from the study.

Informed consent was taken from all patients and the study was approved by the local ethical committee.

### Echocardiographic evaluation

Two dimensional transthoracic echocardiographic and Doppler studies were performed at the time of admission of all patients using the commercially available (M5S probe, GE Vivid E9 echocardiographic system) with a 2.5 MHz transducer.

#### LV function

The biplane method of discs was used to measure LVEF. From the trans-mitral flow profile, trans-mitral pulsed-wave Doppler was obtained, and the peaks of both early diastolic filling (E) and late diastolic filling (A) were measured. In the apical four-chamber view tissue Doppler imaging (TDI) of the mitral annulus was performed by placing the1- to 2-mm sample volume over the septal mitral valve annulus. The value of è was measured and E/è was calculated. The Maximal LA Volume was measured in the apical 4-chamber view at the ventricular end-systolic frame just before the mitral valve opening from the apical views. LA volumes were indexed to the body surface area (LAVI). [22].

#### RV function

TAPSE was calculated in 2-dimensional M-mode echocardiograms from the 4-chamber view by positioning the M-mode cursor on the lateral tricuspid annulus and calculating the amount of longitudinal displacement of the annulus at peak systole [23].

Using the peak velocity (Vmax) of the tricuspid regurgitation Continuous-wave Doppler tracing, the pulmonary artery systolic pressure (PSAP) was determined as the difference in pressures between the right ventricle and the right atrium. The simplified Bernoulli equation (PSAP = 4(V_max_)2 + right atrial pressure) was used [24].

Right atrial pressure (RAP) was derived based on the size and distensibility of the inferior vena cava (IVC) during respiration. IVC diameter of 2.1 cm collapsed > 50% with a sniff indicated normal RA pressure of 3 mmHg, but IVC diameter > 2.1 cm collapsed 50% with a sniff indicated high RAP of 15 mmHg. If the IVC diameter and collapse did not suit this paradigm, an 8-mmHg intermediate value was employed [25].

RV-PA coupling was calculated using the ratio between TAPSE and PASP (TAPSE/PASP) [26].

Right Ventricle Fraction area change (RVFAC) was obtained by tracing the RV end-diastolic area (RVEDA) and end-systolic area (RVESA) in the apical 4-chamber view using the formula (RVEDA − RVESA)/RVEDA × 100[23].

The RA Volume was measured using the 4-chamber at the ventricular end-systolic frame just before the tricuspid valve opening from the apical views. Right atrial volume index (RAVI) was indexed to the body surface area. [27].

The severity of tricuspid regurgitation was also evaluated in from the apical four-chamber view and assessed semiquantitatively from the Color Doppler Flow (mild degree: up to 1/3 of the right atrium (RA), moderate degree: 1/3–2/3 of RA, severe degree: 2/3–the full length of RA) [28].

An experienced echocardiographer carried out all the measurements.

Intraobserver and interobserver variability were assessed n 15 randomly selected patients by repeated analysis on the same cine loop by the same investigator or independently by two separate investigators using intraclass correlation coefficient.

#### Laboratory evaluation

All patients had a measurement of B-type natriuretic peptide (BNP), hemoglobin level, serum sodium, serum potassium, glucose, C-Reactive Protein (CRP), creatinine, bilirubin, AST (aspartate aminotransferase), and ALT (Alanine aminotransferase).

All clinical, demographic, echocardiographic data and laboratory investigations were evaluated at the time of admission.

### Statistical analysis

Statistical studies were carried out using analyzed using IBM SPSS software package version 20.0. (Armonk, NY: IBM Corp). The quantitative variables are expressed as mean + standard deviation (SD). The qualitative data are expressed as counts and percentages. Verification of normality of distribution was performed using The Kolmogorov–Smirnov test.

To compare qualitative values chi-square (Fisher or Monte Carlo) was used. Student t-test was used to compare two groups for normally distributed quantitative variables. Mann Whitney test was used to compare two groups for not normally distributed quantitative variables. Univariate and multivariable logistic regression analyses were performed to detect independent predictors of in-hospital mortality. Receiving operator characteristics (ROC) curve is used to detect optimal cut-off values of TAPSE/PASP for predicting in-hospital mortality. A *p*-value < 0.05 is considered statistically significant.

In addition, the power of the sample size was calculated by the G Power tool (Franz Faul, University of Kiel, Germany, version 3.1.9.4) with 0.05 alpha and 0.6 effect size. The calculated power value was 0.86 according to to post hoc-type power analysis.

## Results

The study cohort included two hundred consecutive patients who were hospitalized for a diagnosis of acute decompensation of chronic heart failure. Patients were divided into two groups: the in-hospital mortality group (n = 24 [12%], and the Survival group (n = 176 [88%]).

### Baseline clinical, hemodynamic, and laboratory characteristics

The baseline clinical characteristics hemodynamic and laboratory characteristics are shown in Tables 1 and 2. There were no significant differences between both groups regarding age, sex, body mass index, NYHA class prior to decompensation, length of stay in the intensive care unit, length of hospital stay, heart failure etiology, hypertension, dyslipidemia, diabetes mellitus, smoking status, atrial fibrillation, stroke, peripheral vascular disease, chronic obstructive pulmonary disease or asthma, major medication prior to decompensation, the incidence of implantation of Implantable Cardioverter Defibrillator (ICD)/permanent pacemaker, need for the vasodilator, non-invasive ventilation, diastolic blood pressure, heart rate, oxygen saturation, hemoglobin concentration and levels of sodium, potassium, Glucose, CRP, bilirubin, AST, ALT. Patients in the in-hospital mortality group were older, with more previous admission due to heart failure excerption, had a longer hospital stay, and had a higher need for vasopressors, inotropes, invasive ventilation, lower systolic blood pressure (mmHg), lower oxygen saturation, higher CRP level, higher Serum creatinine and BNP levels (*p*-value = 0.005, < 0.001, < 0.001, 0.001, 0.001, 0.001, < 0.001, 0.036, < 0.001, and < 0.001 respectively).

**Table 1:** Baseline clinical characteristics of the studied groups

	In-hospital mortality (n = 24)	Survival (n = 176)	*p*
Age (years)	59.71 ± 5.55	55.90 ± 7.39	0.005
Male	14 (58.3%)	99 (56%)	0.847
Body mass index (kg/m^2^)	25.96 ± 1.76	25.68 ± 1.74	0.458
*NYHA class prior to decompensating*			
I	2 (8%)	24 (13%)	0.562
II	2 (8%)	32 (18%)	
III	12 (50%)	72 (40%)	
IV	8 (33%)	48 (27%)	
Length of hospital stay	9.46 ± 2.78	6.50 ± 1.67	< 0.001
(ICD)/permanent pacemaker	3 (12.5%)	17 (9.7%)	0.715
Number of previous AHF admission	2.04 ± 1.08	1.01 ± 0.70	< 0.001
*Heart failure etiology*			
Ischemic heart disease	7 (29%)	56 (32%)	0.632
Dilated cardiomyopathy	10 (42%)	80 (45%)	
Hypertensive	4 (16%)	30 (17%)	
Valvular heart disease	3 (12%)	10 (6%)	
*Comorbidities*			
Hypertension	5 (21%)	33 (19%)	0.784
Dyslipidemia	6 (25%)	42 (24%)	0.903
Diabetes mellitus	4 (17%)	33 (19%)	1.000
Smoker	5 (21%)	28 (16%)	0.559
Atrial fibrillation	8 (33%)	58 (32%)	0.970
Stroke	2 (8%)	11 (19%)	0.658
Chronic obstructive pulmonary disease or asthma	2 (8%)	13(7%)	0.697
Peripheral vascular disease	3 (12%)	16 (29%)	0.708
*Medication prior to decompensating*			
Beta-blocker	7 (29%)	60 (34%)	0.632
ACEI/ARB	13 (54%)	95 (53%)	0.986
Loop diuretic	20 (83%)	149 (85%)	0.771
MRA	5 (21%)	34 (19%)	0.790
Digoxin	7 (29%)	60 (34%)	0.632
Sacubitril/valsartan	3 (12.5%)	19 (10.8%)	0.733
SGLT2 inhibitors	4 (16.7%)	22 (12.5%)	0.526
*Intensive care unit therapies*			
Vasopressors	18 (75%)	35 (20%)	< 0.001
Inotropes	16 (67%)	17(10%)	< 0.001
Vasodilators	6 (25%)	46(26%)	0.905
Invasive ventilation	7 (29%)	5 (3%)	< 0.001^*^
Noninvasive ventilator	13 (54%)	63 (20%)	0.082

*ACEI* Angiotensin-converting enzyme inhibitor; *ARB* Angiotensin II receptor blocker; *MRA* Mineralocorticoid receptor antagonist; *AHF* Acute Heart Failure; *ICD* Implantable cardioverter defibrillator, *SGLT2* Sodium-glucose Cotransporter-2

**Table 2:** Hemodynamic and laboratory characteristics of the studied groups

	In-hospital mortality (n = 24)	Survival (n = 176)	*p*
*Hemodynamics*			
SBP (mmHg)	114.0 ± 10.91	120.7 ± 8.94	0.001
DBP (mmHg)	69.29 ± 4.88	70.03 ± 4.68	0.472
Heart rate (beat/min)	92.13 ± 3.04	92.89 ± 5.98	0.325
Oxygen saturation%	91.83 ± 2.30	94.65 ± 1.73	< 0.001
*Biochemistry*			
BNP (pg/ml)	805 ± 121	596 ± 87.7	< 0.001
Hemoglobin (g/L)	11.29 ± 1.43	11.25 ± 1.41	0.892
Sodium (mmol/L)	137.63 ± 11.21	138.40 ± 3.26	0.740
Potassium (mmol/L)	5.01 ± 0.63	4.92 ± 0.59	0.689
Glucose (mg/dL)	155.0 ± 35.41	147.6 ± 31.59	0.285
CRP (g/L)	28.95 ± 9.03	24.78 ± 5.1	0.036
Serum creatinine (mg/dL)	1.70 ± 0.42	1.23 ± 0.32	< 0.001
Bilirubin (mg/dL)	0.68 ± 0.14	0.66 ± 0.14	0.453
AST (U/L)	22.0 ± 3.89	21.10 ± 1.90	0.277
ALT (U/L)	23.50 ± 4.10	22.64 ± 2.08	0.323

*SBP* Systolic blood pressure; *DBP* Diastoilic blood pressure; *CRP* c reactive protein; *AST* Aspartate aminotransferase; *ALT* Alanine aminotransferase, *BNP* B-type natriuretic peptide

### Echocardiographic characteristics

- Left ventricle function echocardiography study (Table 3): Both groups did not differ with respect to peak mitral E wave velocity, peak mitral A wave velocity LV IVRT, mitral E/e′ ratio, and LAVI. LVEF% was lower in the in-hospital mortality group.
- RV function (Table 4): There were no significant differences between both groups regarding peak tricuspid E wave velocity and peak tricuspid A wave velocity, tricuspid E/è, RVFAC, and severity of tricuspid regurgitation. The pulmonary artery systolic pressure (PASP) and RAVI were higher and TAPSE, TAPSE/PASP (mm/mmHg) were lower in the in-hospital mortality group (*p*-value 0.010, < 0.001, 0.009, and 0.005 respectively).

**Table 3:** The LV echocardiographic characteristics of the studied groups

	In-hospital mortality (n = 24)	Survival (n = 176)	*p*
EF%	31.75 ± 3.95	35.70 ± 3.15	< 0.001
Mitral Peak E (m/s)	0.74 ± 0.10	0.75 ± 0.11	0.644
Mitral Peak A (m/s)	0.80 ± 0.12	0.77 ± 0.11	0.235
LV IVRT (ms)	91.54 ± 20.31	86.62 ± 15.05	0.262
Mitral E/ è	11.21 ± 1.72	11.76 ± 1.78	0.153
LAVI (mL/m^2^)	38.42 ± 6.11	36.84 ± 5.44	0.191

*EF*% Ejection fraction; *E* Peak flow velocity during the early rapid filling phase; *A* Peak flow velocity during atrial contraction; *IVRT* Isovolumic relaxation time; *E/è* The ratio of early flow velocity to the early annular velocity. *LAVI* Left Atrial Volume Index

**Table 4:** The RV echocardiographic characteristics of the studied groups

	In-hospital mortality (n = 24)	Survival (n = 176)	*p*
Tricuspid E(m/s)	0.51 ± 0.07	0.48 ± 0.07	0.090
Tricuspid A(m/s)	0.40 ± 0.07	0.40 ± 0.07	0.829
Tricuspid E/ è	4.50 ± 0.93	4.27 ± 0.82	0.199
PASP (mmHg)	48.88 ± 5.97	45.62 ± 5.68	0.010
TAPSE (mm)	17.67 ± 3.57	20.06 ± 4.27	0.009
TAPSE/PASP (mm/mmHg)	0.38 ± 0.09	0.45 ± 0.12	0.005
RVFAC %	34.88 ± 3.83	36.58 ± 4.32	0.068
RAVI (mL/m2)	37.62 ± 6.30	30.95 ± 5.53	< 0.001
*Severity of TR*			
Mild	8 (33.3%)	64 (36.4%)	0.950
Moderate	9 (37.5%)	65 (36.9%)	
Severe	7 (29.2%)	47 (26.7%)	

*E* Peak flow velocity during the early rapid filling phase; *A* Peak flow velocity during atrial contraction. *E/è* The ratio of early flow velocity to the early annular velocity; *PASP* Pulmonary artery systolic pressure; *TAPSE* Tricuspid annular plane systolic excursion; *RVFAC* Right ventricular fraction area change; *RAVI* Right atrial volume index; *TR* Tricuspid regurgitation

Univariate and multivariable logistic regression analyses were built to identify predictors of in-hospital mortality. The results showed that TAPSE/PASP was an independent predictor for in-hospital mortality (odd ratio = 5.0; 95% confidence interval, (1.890–13.230), *p*-value = 0.001) and (odd ratio = 119.868; 95% confidence interval, (1.246–11,530.0), *p*-value = 0.040) in univariate and multivariable logistic regression analyses respectively (Table 5).

**Table 5:** Univariate and Multivariable logistic regression analysis to predict In-hospital mortality

	Univariate		^#^Multivariate	
	***p***	OR (95%CI)	p	OR (95%CI)
Age (years)	0.018*	1.075 (1.012–1.142)	0.757	0.972 (0.812–1.163)
SBP (mmHg)	0.002*	0.921 (0.875–0.970)	0.018*	0.833 (0.716–0.969)
CRP (g/L)	0.001*	1.135 (1.050–1.227)	0.018*	1.377 (1.056–1.796)
Serum creatinine (mg/dL)	< 0.001*	22.515 (6.475–78.287)	0.009*	64.382 (2.855–1451.8)
EF%	< 0.001*	0.715 (0.619–0.826)	0.009*	0.470 (0.266–0.831)
TAPSE/PASP	0.001*	5.0 (1.890–13.230)	0.040*	119.868 (1.246–11,530.0)
Vasopressor	< 0.001*	7.778 (3.086–19.602)	0.012*	17.834 (1.862–170.82)
BNP (pg/ml)	< 0.001*	1.013 (1.009–1.017)	0.004*	1.032 (1.010–1.053)
RAVI (mL/m2)	< 0.001*	1.195 (1.107–1.289)	0.060	1.220 (0.992–1.502)
Number of previous AHF admission	0.018*	3.470 (1.240–9.705)	0.094	47.986 (0.520–4427.93)

^#^: All variables with *p* < 0.05 was included in the multivariate

^*^: Statistically significant at *p* ≤ 0.05

*OR* Odd`s ratio; *CI* Confidence interval; *LL* Lower limit; *UL* Upper Limit *SBP* Systolic blood pressure; *CRP* c reactive; *EF* Ejection fraction; *TAPSE* Tricuspid annular plane systolic excursion; *PASP* Pulmonary artery systolic pressure; *BNP* B-type natriuretic peptide

Also, the need for vasopressor, elevated BNP, CRP, serum creatinine, lower systolic pressure, and EF% were independent predictors of mortality.

**Fig. 1:**
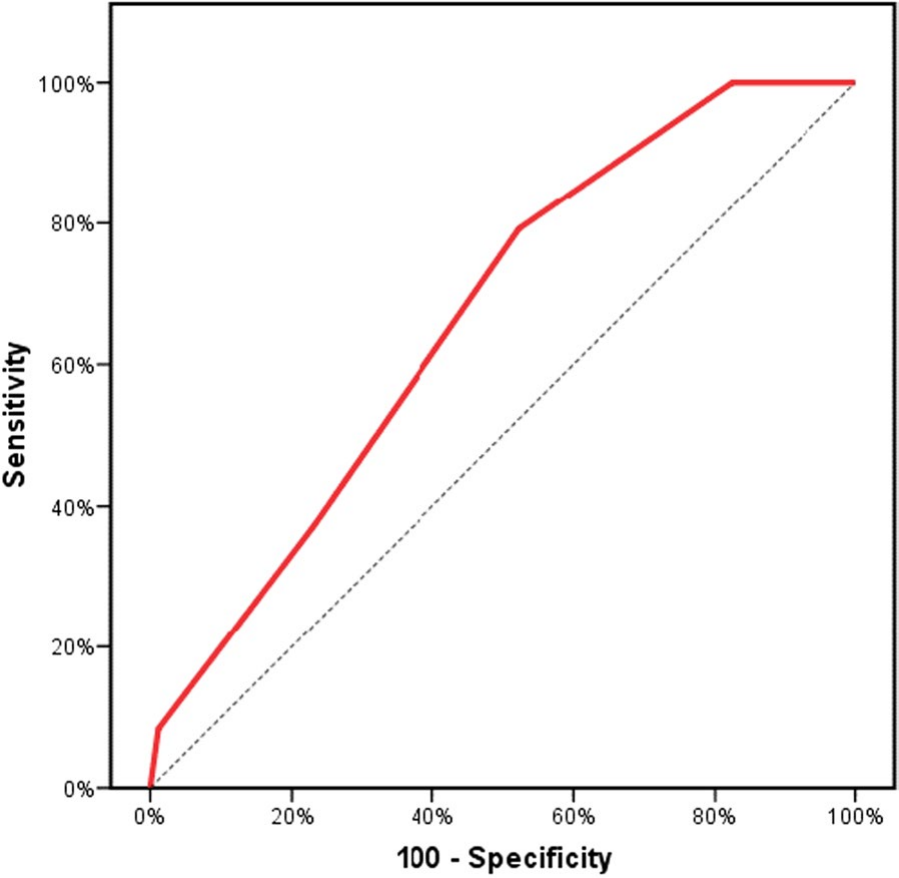
ROC curve for TAPSE/PASP (mm/mmHg) as a predictor for in-hospital mortality

In ROC curve analysis, TAPSE/PASP with a cut-off value < 0.4 mm/mmHg had a sensitivity of 79.17, a specificity of 47.73, and an area under ROC curve = 0.666 for predicting in-hospital mortality (Fig. 1).

Furthermore, patients in each group were classified into three tertiles, tertile1 with TAPSE/PASP < 0.4, tertile2 with TAPSE/PASP between (0.4 & 0.7) and tertile3 with TAPSE/PASP > 0.7. Most of the patients in the mortality group fall within the first tertile 79.2% and there is a statistically significant difference between both groups *p*-value = 0.043. (Table 6).

**Table 6:** TAPSE/PASP ratio tertiles of the study population and cause of death in mortality group

	In-hospital mortality (n = 24)	Survival (n = 176)	*p*
*TAPSE/PASP (mm/mmHg)*			
1st Tertile (< 0.4)	19 (79.2%)	92 (52.3%)	0.043
2nd Tertile (0.4–0.7)	5 (20.8%)	81 (46.0%)	
3rd Tertile (> 0.7)	0 (0%)	3 (1.7%)	
*Cause of death in mortality group*			
VF	4 (16.7%)	–	–
VT	2 (8.3%)	–	–
Asystole	3 (12.5%)	–	–
Renal failure	3 (12.5%)	–	–
Cardiogenic shock	12 (50.0%)	–	–

*VF* Ventricular Fibrillation; *VT* Ventricular Tachycardia; *PASP* Pulmonary artery systolic pressure; *TAPSE* Tricuspid annular plane systolic excursion

Finally, the cause of mortality was ventricular fibrillation (16.7%), ventricular tachycardia (8.3%), asystole (12.5%), renal failure (12.5%), and cardiogenic shock (50.0%) ( Table 6).

### Reproducibility

Intra-observer and inter-observer variability for conventional two-dimensional/Doppler measurements and TDI-derived parameters ranged from 0.94 and 0.97 and 0.92 and 0.94 respectively.

## Discussion

Acute heart failure (AHF) is a leading cause of hospital admissions and is linked with a marked increase in morbid and fatal events [29]. The prognosis of AHF is poor despite advances in therapeutic options with in-hospital mortality rates between 4 and 7% [6].

This prospective cohort study aimed to explore the value of the TAPSE/PASP ratio in predicting in-hospital mortality in patients with acute heart failure in HFrEF patients admitted with acute heart failure.

The main findings of the present study were: (1) The in-hospital mortality of AHF patients was 12%. (2) TAPSE/PASP was an independent predictor for in-hospital mortality in patients with acute decompensation of HFrEF. (3) TAPSE/PASP with a cut-off value < 0.4 mm/mmHg had a sensitivity of 79.17, a specificity of 47.73, and an area under ROC curve = 0.666 for predicting in-hospital mortality (4) The majority of patients in the mortality group (79.2%) had TAPSE/PASP of < 0.4.

The in-hospital mortality in the current study is similar to the finding of Wang HK et al. [30] and close to the mortality rate reported in some large registries as the study submitted by Wajner A et al. [31], in brazil tertiary hospitals.

While in other studies where the number of patients is larger, the in-hospital mortality was slightly lower, like in the study of C. Lombardi et al.[32], who studied 728 patients with AHF, the in-hospital deaths were 8.9%. These differences could be attributed to the differences in the number of patients included in these studies and to the facilities and hearth care quality offered to the patients.

In normal hearts, the synchronization between the RV and pulmonary circulation is important and makes both work as a single cardiopulmonary unit resulting in matching between contractility and afterload, and this is called (RV-PA coupling)[33].

In patients who develop pulmonary hypertension, the pulmonary vascular resistance is increased and the compliance in vascular bed is reduced with increased afterload and alteration in RV-PA coupling resulting in poor outcomes in patients with pulmonary hypertension [34].

Tello et al. proved that TAPSE/PASP is a reliable method for evaluation of RV-PA coupling in patients with severe idiopathic and thromboembolic pulmonary hypertension and, they showed that a value of this ratio < 0.31 mm/mm could be used as a predictor of RV-PA uncoupling [14].

TAPSE/PASP ratio reflects the RV response to changes in the afterload [35] with the higher the ratio, the better the RV function [13] with maintained RV-PA coupling in the initial stages of chronic PH the RV enhances its contractility to counteract the increased afterload, which is frequently accompanied by RV hypertrophy and dilatation. As chronic PH progresses to RV failure, RV-PA uncoupling causes a decline in RV systolic function [36].

The current study adds to the prior reports on the importance of non-invasive echocardiographic measurement of RV-PA coupling in risk stratification in critically ill patients and patients with heart failure.

Jentzer. et al., in their cohort study, found that the loss of RV-PA coupling was linked to increased mortality risk during hospitalization and after discharge among patients admitted to the cardiac intensive care unit [37]. Also, Guazzi. et al. found that TAPSE/PASP was inversely related to NYHA functional class, and they concluded that this ratio which combined measurement of both longitudinal RV fiber shortening (TAPSE) to the developed pressure in the pulmonary artery is a good clinical index that relates the length of RV contraction to the force generated and the use of this ratio is better than the use of both separately and this was vailed for both HFrEF and HFpEF patients [13].

Bragança et al. conducted a retrospective analysis on 70 HF patients who had CRT implantation. TAPSE/PASP ratio demonstrated the best predictive capacity to detect non-response to cardiac resynchronization therapy (CRT [16].

Santas et al. reported that TAPSE/PASP was a predictor of readmission in a prospective study of 1,127 patients with HFpEF fraction discharged with a diagnosis of AHF. Patients in the lowest quintile (TAPSE/PASP < 0.28) had the highest incidence of repeat admissions [19].

The above-mentioned data come with our results that TAPSE/PASP could be used as an independent predictor of in-hospital mortality in patients admitted with acute heart failure.

The association between reduced TAPSE/PASP and mortality in patients with acute decompensation of HFrEF reported in the present study suggests that measures that improve RV-PA coupling may lead to a better outcome. This emphasizes the importance of RV function in patients with LV failure and suggests that optimizing biventricular function in this group of patients is necessary [38].

Therapeutic interventions that improve coupling may have a favorable effect on outcomes. These interventions may include treatments that reduce the RA and pulmonary artery pressure [39]. Such therapeutic interventions might include treatments using either diuretic, drugs that affect the pulmonary vasculature, fluid removal, and short-term inotropic. These options may result in short-term enhancement of RV-PA coupling during the hospital stay and enable recovery from the acute stage [40], 41.

## Conclusions

The non-invasive TAPSE/PASP ratio could be an independent predictor of mortality in HErEF patients presenting with acute heart failure. We recommend further multicentre studies on a larger number of patients to validate our results.

## Study limitations

A relatively small number of patients is a limitation of the current study, also further studies over a longer period of follow-up are recommended as the study analysis was limited to outcomes during hospitalization. Studies with follow-up estimation of TAPSE/PASP and evaluation of the effect of different AHF therapeutic options on RV-PA coupling are required.

## Data Availability

The datasets used and/or analysed during the current study are available from the corresponding author on reasonable request.

## Abbreviations

AHF: Acute heart failure
RV: Right ventricular
PH: Pulmonary hypertension
RVF: Right ventricular failure
HF: Heart failure
TAPSE: Tricuspid annular plane systolic excursion
(Ees/Ea): End-systolic/arterial elastance ratio
RV-PA: Right ventricular-pulmonary artery
HFrEF: HF and reduced ejection fraction
NYHA: New York heart association
LVEF: Left ventricular ejection fraction
ED: Emergency department
E/ è: The ratio of early flow velocity to the early annular velocity
E: Early diastolic filling
A: Late diastolic filling
TDI: Tissue Doppler imaging
Vmax: Peak velocity
RAP: Right atrial pressure
LAVI: Left atrial volume index
IVC: Inferior vena cava
RVFAC: Right ventricular fractional area change
RAVI: Right atrial volume index
RA: Right atrium
HFpEF: Heart failure with preserved
HFmrEF: Heart failure with a mid-range ejection fraction
CRP: C-reactive protein
AST: Aspartate transaminase
ALT: Alanine transaminase
CRT: Cardiac resynchronization therapy
